# Granulomatous Hepatitis Mimicking Hilar Cholangiocarcinoma

**DOI:** 10.4269/ajtmh.24-0850

**Published:** 2025-04-22

**Authors:** Kartik Mehta, Sanjeev Sachdeva, Venkatesh Vaithiyam

**Affiliations:** Department of Gastroenterology, GB Pant Hospital, New Delhi, India

**Figure 1.**
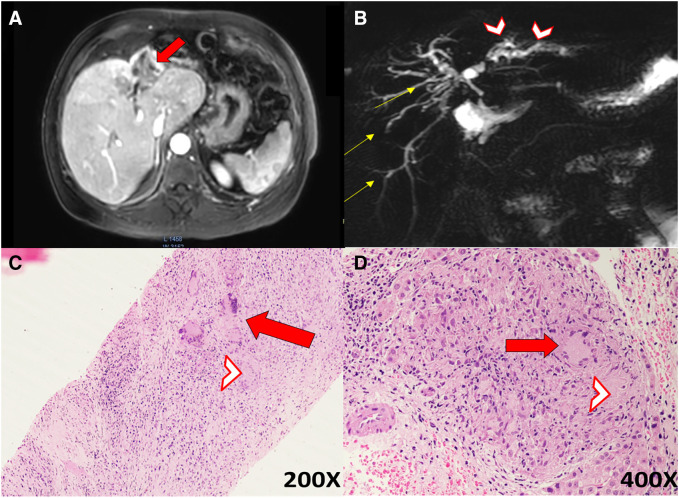
(**A**) Magnetic resonance imaging of the abdomen showing a hypointense mass involving an atrophied left lobe of the liver with faint rim and heterogeneous central enhancement (red arrow). (**B**) Magnetic resonance cholangio pancreatography (MRCP) showing intrahepatic biliary radicles dilatation, predominantly in the left lobe, with abrupt tapering at the hilum suggestive of a stricture. Evidence of an atrophy–hypertrophy complex is seen, with left biliary radicles (arrowheads) being shorter, irregular, and more distended than the long and smooth right biliary radicles (yellow arrows). (**C** and **D**) Liver biopsy showing areas of hepatocytic parenchymal loss with confluent epitheloid cell granulomas admixed with numerous Langhans giant cells (arrows) surrounded by moderate chronic inflammatory cell infiltrate, edema, fibrosis, and necrosis (arrowheads; hematoxylin and eosin staining). (**C**) Magnification: ×200. (**D**) Magnification: ×400.

A 50-year-old male presented with right upper quadrant abdominal pain, anorexia, jaundice, weight loss for 3 months, and fever for 3 days. Examination revealed icterus and mild hepatomegaly. His total bilirubin was 5.2 mg/dL, with direct fraction of 4.3 mg/dL; alkaline phosphatase was 856 IU/mL, but other liver enzymes were normal. His chest X-ray was normal. Abdominal ultrasound revealed an ill-defined heteroechoic lesion measuring about 4 × 3 cm in the left lobe of the liver. Magnetic resonance imaging of the abdomen revealed a hypointense mass involving an atrophied left lobe of the liver and focal intrahepatic biliary radical dilatation with stricture at the hilum and compensatory hypertrophy of right lobe, raising the possibility of cholangiocarcinoma (Figure 1A and B). Ultrasound-guided biopsy of the mass was performed. Histopathological examination of the biopsy revealed findings suggestive of hepatobiliary tuberculosis (Figure 1C and D). Real-time polymerase chain reaction (target sequences IS6110 and MPB64) for *Mycobacterium tuberculosis* was positive in the biopsy sample. Based on these investigations, the patient was started on antitubercular therapy, and his symptoms improved.

Our patient presented with obstructive jaundice, and imaging findings were suggestive of an atrophy–hypertrophy complex (AHC), a common cause of which is hilar cholangiocarcinoma.^1^ AHC is observed in pathological conditions associated with obstruction of portal venous inflow and biliary or hepatic venous outflow to a portion of the liver. The AHC here was owing to obstruction at the biliary level caused by tuberculosis, which is an unusual cause of this condition.^2^ Because the clinical and imaging findings of biliary tuberculosis are challenging to differentiate from other causes of AHC, particularly cholangiocarcinoma, histology and molecular testing are the key to diagnosis. Hepatobiliary tuberculosis is a frequent cause of granulomatous hepatitis in developing countries.^3^ Other causes of granulomatous hepatitis include sarcoidosis, primary biliary cholangitis, fungal infections, and drug-induced liver injury.^4^
